# Efficacy of neoadjuvant, adjuvant, and perioperative immunotherapy in non-small cell lung cancer across different PD-L1 expression levels: a systematic review and meta-analysis

**DOI:** 10.3389/fimmu.2025.1569864

**Published:** 2025-05-20

**Authors:** Zhenlong Zhang, Yuchen Lin, Shuchen Chen

**Affiliations:** ^1^ Department of Thoracic Surgery, Fujian Medical University Union Hospital, Fujian Medical University, Fujian, China; ^2^ Department of Thoracic Surgery, Shengli Clinical Medical College of Fujian Medical University, Fujian Provincial Hospital, Fujian, China

**Keywords:** immunotherapy, efficacy, non-small cell lung cancer, PD-L1 expression, meta-analysis

## Abstract

**Background:**

Immune checkpoint inhibitors, particularly anti-PD-1/PD-L1 monoclonal antibodies, have transformed non-small cell lung cancer (NSCLC) treatment. This meta-analysis evaluates the efficacy of neoadjuvant, adjuvant, and perioperative immunotherapy in resectable NSCLC, stratified by PD-L1 expression levels.

**Methods:**

We conducted a meta-analysis of 10 randomized controlled trials (RCTs) involving 11 articles, focusing on pathological complete response (pCR), major pathological response (MPR), event-free survival (EFS), and overall survival (OS). These outcomes were stratified by PD-L1 expression levels (<1%, ≥1%, 1-49%, ≥50%).

**Results:**

Immunotherapy significantly improved pCR (OR=4.96, 95% CI=2.88–8.57 for PD-L1<1%; OR=9.58, 95% CI=6.32–14.53 for PD-L1≥1%), MPR (OR=2.86, 95% CI=1.97–4.16 for PD-L1<1%; OR=7.39, 95% CI=4.59–11.88 for PD-L1≥1%), and EFS (HR=0.80, 95% CI=0.70–0.92 for PD-L1<1%; HR=0.53, 95% CI=0.45–0.62 for PD-L1≥1%) across all PD-L1 subgroups. Greatest benefits were observed in PD-L1≥50% subgroup, with ORs for pCR and MPR, and HRs for EFS, showing consistent improvements. OS benefits were significant in PD-L1≥1% patients (HR=0.62, 95% CI=0.49–0.79 for PD-L1≥1%) but uncertain in PD-L1<1% cohorts (HR=1.11, 95% CI=0.86–1.44 for PD-L1<1%). Immunotherapy in perioperative setting demonstrated robust efficacy, with significant pathological response and EFS benefits across all PD-L1 subgroups.

**Conclusion:**

This meta-analysis supports immunotherapy within perioperative care for resectable NSCLC, emphasizing PD-L1 expression as a predictive biomarker. Future studies should optimize patient selection and clarify immunotherapy’s role in different treatment settings.

**Systematic review registration:**

https://www.crd.york.ac.uk/PROSPERO/view/CRD42025644497, identifier CRD42025644497.

## 1 Introduction

In 2022, lung cancer remained the leading cause of global cancer incidence (12.4%) and mortality (18.7%) (1), with non-small cell lung cancer (NSCLC) comprising 80%-85% of cases (2). While surgery is the mainstay for early- and intermediate-stage NSCLC (3), 52%-75% of stage II-III patients experience recurrence or metastasis within five years (4). Adjuvant and neoadjuvant chemotherapy provide only a modest 5% survival benefit, highlighting the need for more effective strategies (5, 6). The advent of immune checkpoint inhibitors, particularly anti-PD-1/PD-L1 monoclonal antibodies, has transformed NSCLC treatment, shifting immunotherapy from advanced to early- and intermediate-stage use. Recent phase III trials have demonstrated that neoadjuvant, adjuvant, and perioperative “sandwich” immunotherapy significantly reduce recurrence risks, establishing these approaches as standard for stage II-III NSCLC. However, challenges persist in patient selection, regimen optimization, and perioperative management.

Currently, there is no definitive predictive biomarker to guide the integration of immunotherapy with surgical resection in NSCLC. In the neoadjuvant setting, studies such as CheckMate-816, AEGEAN, CheckMate-77T, and RATIONALE-315 have explored the relationship between PD-L1 expression levels and pathological responses, demonstrating improved pathological complete response (pCR) and major pathological response (MPR) rates across varying PD-L1 expression levels (6–10). Although patients with high PD-L1 expression may derive greater pathological and survival benefits from neoadjuvant immunotherapy, neither the FDA nor NMPA mandates PD-L1 testing for approved neoadjuvant immunotherapy indications in NSCLC. In the adjuvant setting, the KEYNOTE-091 trial showed significant disease-free survival (DFS) benefits with pembrolizumab, but the trend was less clear in the PD-L1≥50% subgroup (11). Conversely, the IMpower010 trial revealed that atezolizumab provided the most substantial DFS and overall survival (OS) benefits in patients with PD-L1≥50% (12, 13). These conflicting findings underscore the ongoing debate regarding the optimal PD-L1 threshold for incorporating immunotherapy and how to tailor treatment choices—neoadjuvant, adjuvant, or perioperative—based on PD-L1 expression levels.

Further research is essential to elucidate the efficacy of immunotherapy across different treatment settings and PD-L1 expression subgroups, aiming to maximize clinical benefits. Previous meta-analysis has explored the impact of PD-L1 expression on outcomes in surgical NSCLC patients receiving immunotherapy, but these were largely limited to small-sample or single-arm studies (14). To address this gap, our study leverages high-quality evidence from randomized controlled trials (RCTs) to systematically evaluate the benefits of incorporating neoadjuvant, adjuvant, and perioperative immunotherapy in NSCLC patients stratified by PD-L1 expression levels.

## 2 Methods

This study was registered in PROSPERO (CRD42025644497) and conducted in accordance with the PRISMA guidelines (15). The PICOS framework for this meta-analysis is as follows:

Participants: Patients with histologically confirmed resectable NSCLC.
Intervention: Use of immunotherapy (anti-PD-1/PD-L1 agents) in the neoadjuvant, adjuvant, or perioperative setting, either alone or in combination with chemotherapy.
Control: Placebo (in double-blind RCTs), no placebo (in open-label studies), or best supportive care (BSC).
Outcomes: pCR, MPR, event-free survival (EFS), DFS, progression-free survival (PFS), and OS, stratified by PD-L1 expression levels (<1%, ≥1%, 1-49%, ≥50%).
Study design: RCTs.

### 2.1 Database searching

A comprehensive literature search was conducted in Medline (via PubMed), Web of Science, Embase, and the Cochrane Library up to January 31, 2025. Search terms were designed to capture relevant studies using the following keywords: lung, NSCLC, and non-small cell lung cancer to identify NSCLC-related studies; surgery, resection, segmentectomy, lobectomy, surgical, pneumonectomy, and thoracotomy to limit results to surgical resection; and immunotherapy, immune checkpoint inhibitor, anti PD-1, anti PD-L1, durvalumab, nivolumab, pembrolizumab, toripalimab, tislelizumab, and camrelizumab to specify immunotherapy-related studies. Additionally, the reference lists of eligible articles were manually reviewed to identify further relevant publications.

### 2.2 Eligibility criteria

#### 2.2.1 Inclusion criteria

(1) Histologically confirmed NSCLC; (2) Enrollment of adult patients (≥18 years); (3) RCTs, either phase II or III (4); Investigation of immunotherapy as neoadjuvant, adjuvant, or perioperative treatment, with or without chemotherapy; (5) Reporting of at least one of the following outcomes: pCR, MPR, EFS, DFS, PFS, or OS.

#### 2.2.2 Exclusion criteria

(1) Duplicate records; (2) Non-English publications; (3) Lack of outcome stratification by specific PD-L1 expression levels (<1%, ≥1%, 1-49%, ≥50%); (4) Meta-analyses, reviews, case reports, conference abstracts, letters, animal studies, and single-arm studies.

### 2.3 Study selection

Two experienced investigators independently screened the literature according to the predefined eligibility criteria. Discrepancies in screening results were resolved through consultation with a third investigator. Retrieved records were managed using EndNote X9, and duplicate publications were identified and removed using the software’s deduplication function, followed by manual verification. Initial screening was performed by reviewing titles and abstracts. For potentially eligible studies, full-text articles were assessed to confirm inclusion.

### 2.4 Data extraction

Two investigators independently extracted data, with discrepancies resolved through consultation with a third investigator. The extracted study characteristics included author, publication year, sample size, participant details (e.g., disease stage, PD-L1 expression levels), intervention specifics (e.g., type of immunotherapy, treatment setting), comparator details (e.g., placebo, BSC), and outcome measures. For dichotomous outcomes such as pCR and MPR, the number of events and the total sample size were recorded. For time-to-event outcomes, hazard ratios (HRs) and their corresponding 95% confidence intervals (CIs) were extracted.

### 2.5 Outcomes

The primary outcomes pooled in this meta-analysis included pCR, MPR, EFS, and OS. pCR was defined as the absence of residual viable tumor cells in the resected specimen following neoadjuvant therapy. MPR was defined as ≤10% residual viable tumor cells in the surgical specimen. EFS was defined as the time from randomization to the occurrence of any event, including progressive disease precluding surgery, unresectable tumor, local or distant recurrence, or death from any cause. While some studies reported DFS or PFS instead of EFS, the definitions of these endpoints overlap, with EFS being the most comprehensive. Therefore, studies reporting DFS or PFS were included and analyzed alongside EFS in this meta-analysis.

### 2.6 Risk of bias assessment

The risk of bias of included RCTs was assessed using the Cochrane Risk of Bias Tool (version 2.0) (16). This tool evaluates bias across five domains: (1) bias arising from the randomization process, (2) bias due to deviations from intended interventions, (3) bias due to missing outcome data, (4) bias in the measurement of the outcome, and (5) bias in the selection of the reported result. Each domain was rated as “low risk,” “some concerns,” or “high risk” based on predefined criteria.

### 2.7 Statistical analysis

Statistical analyses were performed using the *meta* package in R (version 4.3.0). The primary pooled analyses of outcomes were conducted separately for four PD-L1 expression subgroups: <1%, ≥1%, 1-49%, and ≥50%. For dichotomous outcomes (pCR, MPR), odds ratios (ORs) with 95% CIs were calculated, with OR >1 favoring the experimental group (immunotherapy). For time-to-event outcomes (EFS, OS), HRs with 95% CIs were pooled, with HR<1 favoring the experimental group. Heterogeneity was assessed using the I² statistic. A random-effects model was applied for data synthesis when I² >50%, indicating substantial heterogeneity; otherwise, a fixed-effects model was used. Sensitivity analyses were performed by sequentially excluding one study at a time to evaluate the robustness of the results. Subgroup analyses were conducted based on the use of placebo, the proportion of squamous histology, and the proportion of patients with ECOG PS=0. Publication bias was assessed using Egger’s test and funnel plots. Cumulative meta-analysis was performed based on the chronological order of study publication. Additionally, separate pooled analyses of outcomes were conducted for studies focusing on neoadjuvant, perioperative, and adjuvant immunotherapy settings.

## 3 Results

### 3.1 Characteristics of included studies and risk of bias

**Figure 1:**
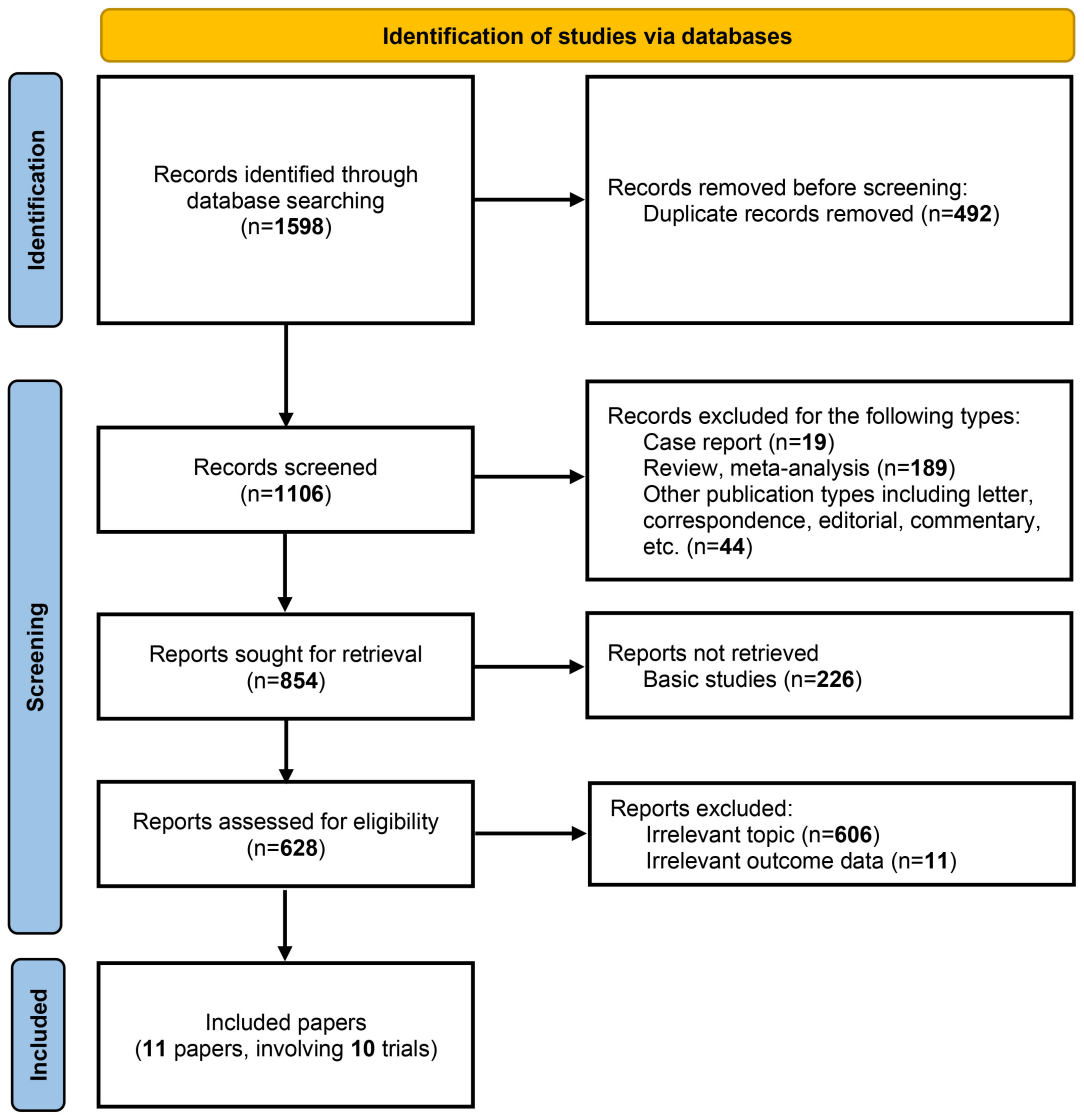
PRISMA flow diagram for study selection. The flow diagram illustrates the systematic process of identifying, screening, and selecting studies for inclusion in the meta-analysis. A total of 1,598 records were identified through database searching. After removing 492 duplicate records, 1,106 records were screened for eligibility. Of these, 854 reports were sought for retrieval, with 226 basic studies excluded. Following a detailed assessment of 628 reports, 11 papers involving 10 trials were ultimately included in the analysis. The exclusion criteria at each stage are detailed in the flow diagram.

**Figure 2:**
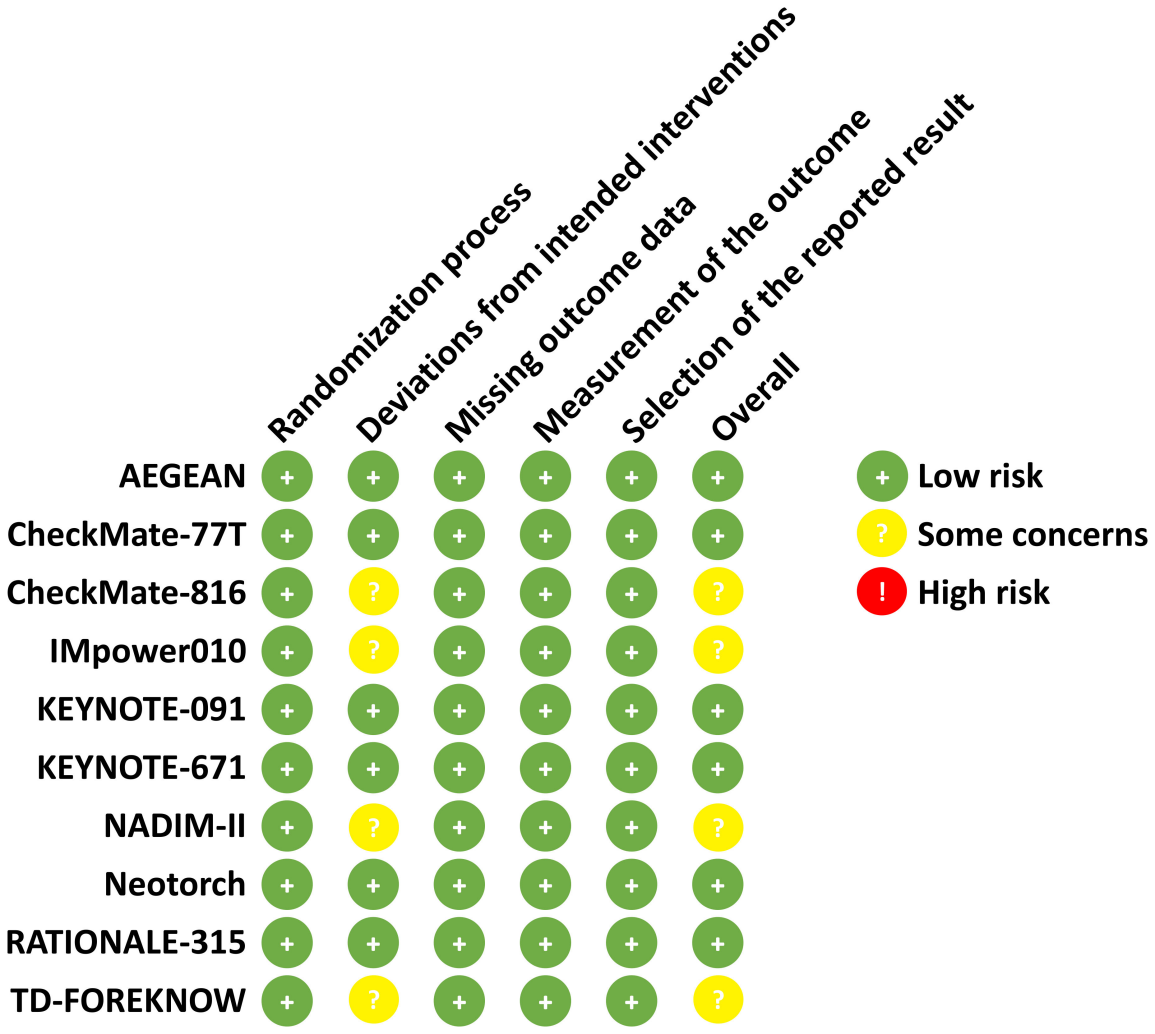
Risk of bias assessment according to the cochrane risk of bias tool (2.0). The risk of bias for each included study is assessed by using the Cochrane Risk of Bias Tool (2.0). The domains assessed include the randomization process, deviations from intended interventions, missing outcome data, measurement of the outcome, and selection of the reported result. Each domain is rated as low risk (+), some concerns ()?, or high risk ()! based on specific criteria.

As illustrated in **Figure 1**, a total of 11 articles with 5569 patients (representing 10 trials) were included after systematic literature search and screening (7–13, 17–20). The characteristics of the included studies are summarized in **Table 1**. The numbers of patients with PD-L1 <1% and ≥1% were 2167 and 3402, respectively. Except for the NADIM-II and TD-FOREKNOW trials, which were phase II studies, all others were phase III RCTs. Two studies evaluated immunotherapy in the neoadjuvant setting, two in the adjuvant setting, and the remainder in the perioperative setting. The immunotherapeutic agents investigated included durvalumab, nivolumab, atezolizumab, pembrolizumab, toripalimab, tislelizumab, and camrelizumab. Control arms predominantly used placebo or no treatment, with the exception of IMpower010, which employed BSC. As shown in **Figure 2**, no studies were rated as high risk of bias, although four studies raised some concerns, primarily due to the absence of blinding. Overall, the quality of the included studies was high, with minimum risk of bias.

**Table 1:** Characteristics of included studies.

Study name	Year	Phase of study	NSCLC stage	Experimental group sample size	Control group sample size	Immunotherapy setting	Experimental group neoadjuvant treatment	Control group neoadjuvant treatment	Experimental group adjuvant treatment	Control group adjuvant treatment	Available outcomes
AEGEAN (9)	2023	3	IIA-IIIB	366	374	Perioperative	Durvalumab +chemotherapy	Placebo +chemotherapy	Durvalumab	Placebo	pCR, MPR, EFS
CheckMate-77T (7)	2024	3	IIA-IIIB	229	232	Perioperative	Nivolumab +chemotherapy	Placebo +chemotherapy	Nivolumab	Placebo	pCR, MPR, EFS
CheckMate-816 (10)	2022	3	IB-IIIA	179	179	Neoadjuvant	Nivolumab +chemotherapy	Chemotherapy	None	None	pCR, MPR, EFS
IMpower010 (12, 13)	2021	3	IB-IIIA	507	498	Adjuvant	None	None	Chemotherapy +atezolizumab	Chemotherapy +best supportive care	DFS, OS
KEYNOTE-091 (11)	2022	3	IB-IIIA	590	587	Adjuvant	None	None	Pembrolizumab	Placebo	DFS
KEYNOTE-671 (17)	2023	3	II-IIIB	397	400	Perioperative	Pembrolizumab +chemotherapy	Placebo +chemotherapy	Pembrolizumab	Placebo	EFS, OS
NADIM-II (18)	2023	2	III	57	29	Perioperative	Nivolumab +chemotherapy	Chemotherapy	Nivolumab	None	PFS, OS
Neotorch (19)	2024	3	II-IIIB	202	202	Perioperative	Toripalimab +chemotherapy	Placebo +chemotherapy	Toripalimab	Placebo	EFS
RATIONALE-315 (8)	2024	3	II-IIIA	226	227	Perioperative	Tislelizumab +chemotherapy	Placebo +chemotherapy	Tislelizumab	Placebo	pCR, MPR, EFS
TD-FOREKNOW (20)	2023	2	IIIA-IIIB	43	45	Neoadjuvant	Camrelizumab +chemotherapy	Chemotherapy	None	None	pCR, MPR

NSCLC, non-small-cell lung cancer; pCR, pathological complete response; MPR, major pathological response; EFS, event-free survival; DFS, disease-free survival; OS, overall survival; PFS, progression-free survival.

### 3.2 Outcomes of immunotherapy across PD-L1 expression subgroups

**Figure 3:**
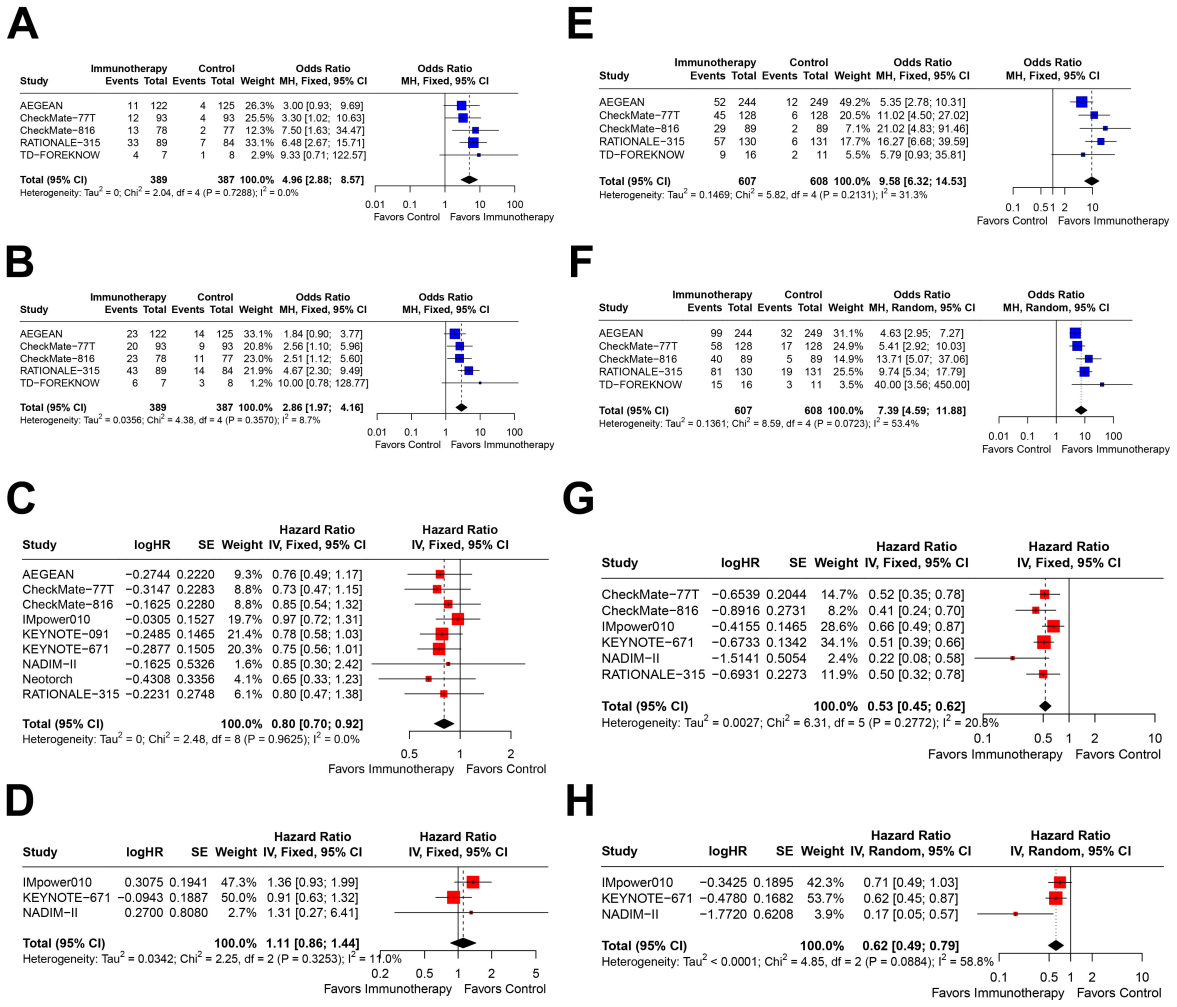
Forest plots depicting the efficacy of immunotherapy across PD-L1 expression levels of <1% and ≥1%. This figure provides forest plots for the outcomes including **(A)** pathological complete response (pCR)rate in patients with PD-L1 expression <1%, **(B)** major pathological response (MPR) rate in patients with PD-L1 expression <1%, **(C)** event-free survival (EFS) in patients with PD-L1 expression <1%, **(D)** overall survival (OS) in patients with PD-L1 expression <1%, **(E)** pCR rate in patients with PD-L1 expression ≥1%, **(F)** MPR rate in patients with PD-L1 expression ≥1%, **(G)** EFS in patients with PD-L1 expression ≥1%, and **(H)** OS in patients with PD-L1 expression ≥1%.

In patients with PD-L1<1%, immunotherapy significantly improved pCR (OR=4.96, 95% CI=2.88–8.57; **Figure 3A**), MPR (OR=2.86, 95% CI=1.97–4.16; **Figure 3B**), and EFS (HR=0.80, 95% CI=0.70–0.92; **Figure 3C**). However, only three studies reported OS outcomes, and no significant benefit was observed in this subgroup (HR=1.11, 95% CI=0.86–1.44; **Figure 3D**).

In the PD-L1≥1% subgroup, immunotherapy demonstrated significant improvements across all four outcomes: pCR (OR=9.58, 95% CI=6.32–14.53; **Figure 3E**), MPR (OR=7.39, 95% CI=4.59–11.88; **Figure 3F**), EFS (HR=0.53, 95% CI=0.45–0.62; **Figure 3G**), and OS (HR=0.62, 95% CI=0.49–0.79; **Figure 3H**). Notably, the effect sizes were consistently greater in this subgroup compared to the PD-L1<1% subgroup.

**Figure 4:**
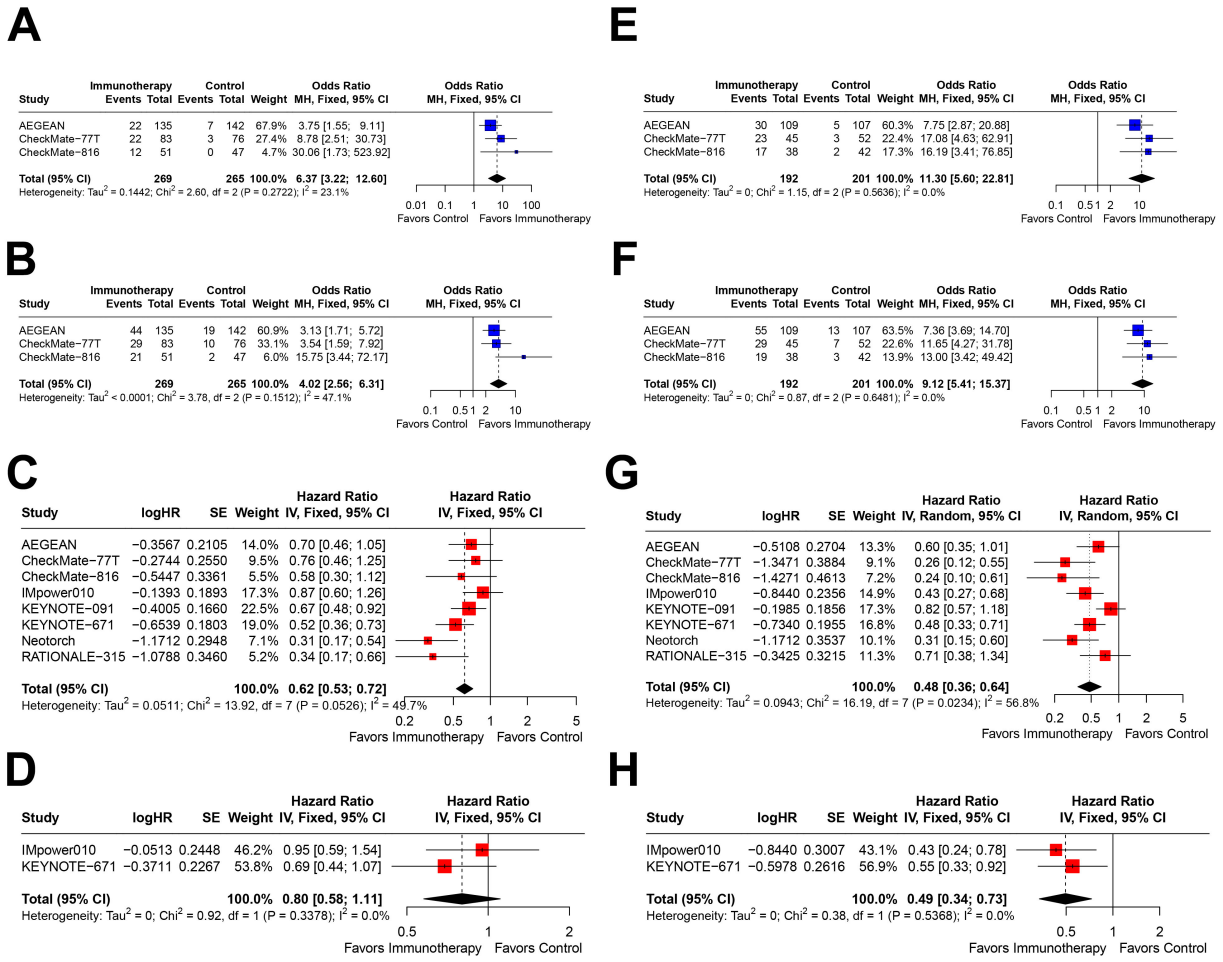
Forest plots depicting the efficacy of immunotherapy across PD-L1 expression levels of 1-49% and ≥50%. This figure provides forest plots for the outcomes including **(A)** pathological complete response (pCR) rate in patients with PD-L1 expression between 1-49%, **(B)** major pathological response (MPR) rate in patients with PD-L1 expression between 1-49%, **(C)** event-free survival (EFS) in patients with PD-L1 expression between 1-49%, **(D)** overall survival (OS) in patients with PD-L1 expression between 1-49%, **(E)** pCR rate in patients with PD-L1 expression ≥50%, **(F)** MPR rate in patients with PD-L1 expression ≥50%, **(G)** EFS in patients with PD-L1 expression ≥50%, and **(H)** OS in patients with PD-L1 expression ≥50%.

Further stratification within the PD-L1≥1% subgroup revealed distinct outcomes. In patients with PD-L1 1–49%, immunotherapy was associated with significant benefits in pCR (OR=6.37, 95% CI=3.22–12.60; **Figure 4A**), MPR (OR=4.02, 95% CI=2.56–6.31; **Figure 4B**), and EFS (HR=0.62, 95% CI=0.53–0.72; **Figure 4C**). However, only two studies reported OS outcomes, which showed a trend toward benefit but did not reach statistical significance (HR=0.80, 95% CI=0.58–1.11; **Figure 4D**).

In contrast, patients with PD-L1≥50% exhibited the most pronounced benefits from immunotherapy, with significant improvements in pCR (OR=11.30, 95% CI=5.60–22.81; **Figure 4E**), MPR (OR=9.12, 95% CI=5.41–15.37; **Figure 4F**), EFS (HR=0.48, 95% CI=0.36–0.64; **Figure 4G**), and OS (HR=0.49, 95% CI=0.34–0.73; **Figure 4H**). Notably, the effect sizes for all outcomes in this subgroup were the most favorable among all PD-L1 expression strata.

### 3.3 Sensitivity analyses

Sensitivity analyses were performed for patients with PD-L1<1% (pCR, **Supplementary Figure S1A**; MPR, **Supplementary Figure S1B**; EFS, **Supplementary Figure S1C**; OS, **Supplementary Figure S1D**), PD-L1≥1% (pCR, **Supplementary Figure S1E**; MPR, **Supplementary Figure S1F**; EFS, **Supplementary Figure S1G**; OS, **Supplementary Figure S1H**), PD-L1 1–49% (pCR, **Supplementary Figure S2A**; MPR, **Supplementary Figure S2B**; EFS, **Supplementary Figure S2C**; OS, **Supplementary Figure S2D**), and PD-L1≥50% (pCR, **Supplementary Figure S2E**; MPR, **Supplementary Figure S2F**; EFS, **Supplementary Figure S2G**; OS, **Supplementary Figure S2H**). The results consistently supported the stability of the primary analysis, as omitting any single study did not significantly alter the overall findings.

### 3.4 Subgroup analysis

Given the greater number of studies reporting EFS, subgroup analyses were conducted for this outcome (**Table 2**). In studies using placebo, significant EFS benefits were observed across all PD-L1 expression strata. In contrast, studies without placebo showed no significant EFS benefits in the PD-L1<1% and 1–49% subgroups, likely due to limited sample size. For studies with squamous NSCLC proportions <50% and ≥50%, significant EFS benefits were consistently observed. Similarly, when stratified by the proportion of patients with ECOG PS=0 (median=62.5%), both subgroups demonstrated significant EFS benefits in all PD-L1 expression strata. Across all subgroups, the pooled HRs for EFS in the PD-L1≥1% subgroup were numerically better than those in the PD-L1<1% subgroup. Furthermore, within the PD-L1≥1% subgroup, the HRs for EFS in the ≥50% stratum were consistently lower than those in the 1–49% stratum.

**Table 2:** Subgroup analysis of EFS.

Subgroup	PD-L1<1%		PD-L1≥1%		PD-L1: 1-49%		PD-L1≥50%	
	No. of studies	HR of EFS, 95%CI	No. of studies	HR of EFS, 95%CI	No. of studies	HR of EFS, 95%CI	No. of studies	HR of EFS, 95%CI
Use of placebo: yes	6	0.756 (0.645-0.886)	3	0.510 (0.419-0.622)	6	0.574 (0.481-0.684)	6	0.522 (0.373-0.730)
Use of placebo: no	3	0.927 (0.728-1.180)	3	0.558 (0.437-0.714)	2	0.789 (0.571-1.090)	2	0.368 (0.222-0.610)
Proportion of patients with squamous NSCLC: <50%	5	0.818 (0.699-0.956)	3	0.554 (0.458-0.670)	4	0.673 (0.562-0.806)	4	0.572 (0.422-0.774)
Proportion of patients with squamous NSCLC: ≥50%	4	0.769 (0.597-0.989)	3	0.485 (0.374-0.629)	4	0.489 (0.364-0.657)	4	0.359 (0.212-0.606)
Proportion of patients with ECOG PS of 0: ≥62.5%	4	0.778 (0.637-0.949)	3	0.577 (0.460-0.724)	4	0.551 (0.437-0.696)	4	0.513 (0.393-0.669)
Proportion of patients with ECOG PS of 0: <62.5%	5	0.825 (0.690-0.986)	3	0.491 (0.399-0.605)	4	0.673 (0.548-0.826)	4	0.440 (0.261-0.741)

EFS, event-free survival; PD-L1, programmed death ligand 1; HR, hazard ratio; CI, confidence interval; NSCLC, non-small-cell lung cancer; ECOG PS, Eastern Cooperative Oncology Group performance-status.

### 3.5 Publication bias assessment

Egger’s test detected no statistically significant publication bias across the four PD-L1 expression subgroups (P=0.511, 0.056, 0.167, and 0.062, respectively). Funnel plots demonstrated good symmetry in the PD-L1<1% subgroup (**Supplementary Figure S3A**), suggesting no publication bias. Slight asymmetry was observed in the PD-L1≥1% (**Supplementary Figure S3B**), 1–49% (**Supplementary Figure S3C**), and ≥50% subgroups (**Supplementary Figure S3D**). Given that PD-L1 stratification is a standard practice in NSCLC immunotherapy RCTs, this minor asymmetry is unlikely to stem from selective reporting. Instead, it may reflect the limited number of studies or inconsistencies in PD-L1 stratification thresholds. For example, while the AEGEAN, KEYNOTE-091, and Neotorch trials reported EFS outcomes for PD-L1 1–49% and ≥50% subgroups, they did not provide data for the PD-L1≥1% subgroup. Conversely, the NADIM-II study reported results for the PD-L1≥1% subgroup but not for the 1–49% and ≥50% subgroups. These variations in reporting practices may contribute to the observed asymmetry.

### 3.6 Cumulative meta-analysis

**Figure 5:**
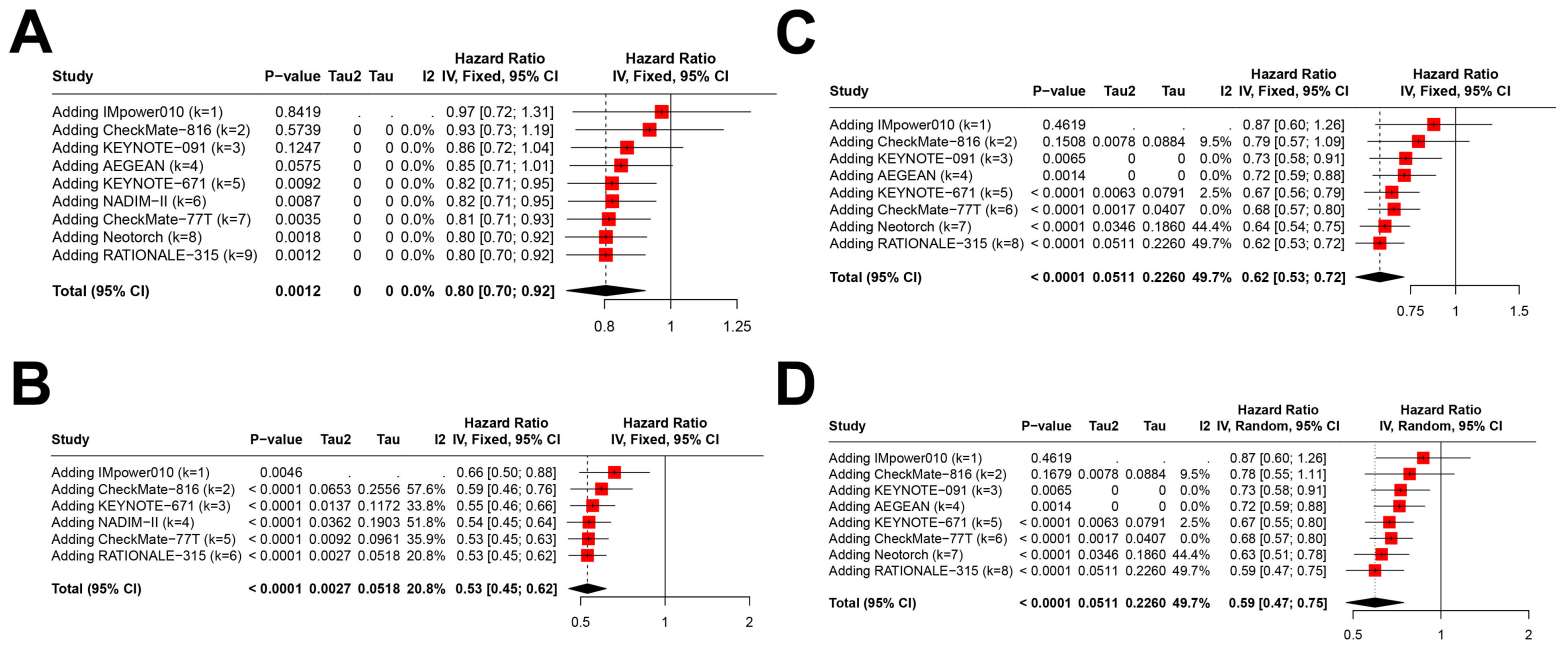
Cumulative meta-analysis of event-free survival (EFS) across different PD-L1 Expression Levels. This figure illustrates the cumulative meta-analysis results over the years for pooled hazard ratios (HRs) of EFS across various PD-L1 expression levels including **(A)** PD-L1 expression <1%, **(B)** PD-L1 expression ≥1%, **(C)** PD-L1 expression between 1-49%, and **(D)** PD-L1 expression ≥50%.

Cumulative meta-analysis demonstrated that the pooled HRs for EFS in the PD-L1<1% subgroup stabilized over time with accumulating evidence (**Figure 5A**), and a similar trend was observed in the PD-L1≥1% subgroup (**Figure 5B**). Notably, the pooled HRs for EFS in the PD-L1 1–49% (**Figure 5C**) and ≥50% subgroups (**Figure 5D**) continued to show a downward trend in recent years, suggesting further refinement of treatment benefits. As previously noted, there were inconsistencies in PD-L1≥1% stratification thresholds across studies. Consequently, the cumulative results for the PD-L1≥1%, 1–49%, and ≥50% subgroups remain to be further validated as more evidence becomes available.

### 3.7 Outcomes in different treatment setting

Although we initially planned to evaluate outcomes by treatment setting (neoadjuvant, perioperative, and adjuvant), the limited number of studies in the neoadjuvant and adjuvant settings (two each) precluded robust subgroup analyses (**Supplementary Table S1**). In the perioperative setting, which included a larger number of studies, the pathological response and EFS results aligned with the overall findings. Significant benefits across all PD-L1 subgroups were observed, with numerically higher ORs and lower HRs in the PD-L1≥1% subgroup compared to the PD-L1<1% subgroup. Furthermore, within the PD-L1≥1% subgroup, the ORs for pCR/MPR and HRs for EFS in the ≥50% stratum suggested consistently more benefits than those in the 1–49% stratum. However, the assessment of OS in the perioperative setting was limited due to the small number of studies reporting this outcome.

## 4 Discussion

This meta-analysis demonstrates that immunotherapy significantly improves pCR, MPR, and EFS across all PD-L1 expression subgroups in NSCLC, including patients with PD-L1<1%. While OS benefits are well-established in PD-L1≥1% populations, the impact on OS in PD-L1<1% patients remains uncertain. Importantly, the benefit increases incrementally with higher PD-L1 expression, with the greatest improvements observed in PD-L1≥50% subgroups. Although perioperative immunotherapy shows robust efficacy, the limited number of studies in neoadjuvant and adjuvant settings underscores the need for further research to define its role across different treatment paradigms. These findings support the integration of immunotherapy into perioperative care while highlighting the importance of PD-L1 expression as a predictive biomarker for treatment response.

The relationship between PD-L1 expression and immunotherapy efficacy has been a focal point of research, particularly in the neoadjuvant setting. Consistent with the recent Chinese expert consensus on perioperative immunotherapy for NSCLC (21), this meta-analysis confirms that pathological responses (pCR and MPR) and EFS benefits are observed across all PD-L1 expression levels, including PD-L1<1% patients. However, a correlation exists between higher PD-L1 expression and greater clinical benefits, with PD-L1-high patients demonstrating the most pronounced improvements in pCR, MPR, and EFS. Specifically, the pooled ORs for pCR and MPR, as well as the HRs for EFS, consistently improved with increasing PD-L1 expression levels, supporting the consensus recommendation for PD-L1 testing when feasible. These findings underscore the utility of PD-L1 as a predictive biomarker, guiding clinicians in identifying patients who are most likely to derive significant benefits from neoadjuvant immunotherapy. Nevertheless, the observed benefits in PD-L1-low and PD-L1<1% subgroups highlight the potential of immunotherapy as a broad therapeutic strategy, even in the absence of high PD-L1 expression.

The traditional rationale for neoadjuvant immunotherapy has been to reduce tumor burden, minimize micrometastases, and facilitate surgical resection (22, 23). However, the emergence of phase III trials exploring the “neoadjuvant + adjuvant” perioperative immunotherapy paradigm has shifted the focus toward long-term survival benefits rather than transient disease control. Despite this progress, the necessity of adjuvant immunotherapy, particularly following neoadjuvant treatment, remains controversial. The predictive value of PD-L1 expression in the adjuvant setting is unclear, as evidenced by conflicting results from the IMpower-010 and KEYNOTE-091 trials. IMpower010 demonstrated a clear DFS benefit in PD-L1≥50% patients (HR=0.43, 95%CI: 0.27–0.68), while KEYNOTE-091 showed no significant DFS benefit in this subgroup (HR=0.82, 95%CI: 0.57–1.18). In our meta-analysis, pooled DFS results from these studies also failed to show a statistically significant benefit in the PD-L1≥50% subgroup, though the HR was numerically lower than in PD-L1<1% patients (**Supplementary Table S1**). Notably, the PD-L1≥50% subgroup represented the smallest proportion of patients in both trials, suggesting that the lack of significance may be due to limited sample size. Future studies with larger cohorts may clarify this issue.

Our analysis pooled data from studies reporting EFS or DFS as endpoints. While these measures differ in scope—EFS includes preoperative events (e.g., unresectability or progression prior to surgery), whereas DFS focuses on postoperative recurrence or death—both are validated surrogates for OS in resectable NSCLC and share a common clinical objective: evaluating long-term disease control. This approach aligns with prior meta-analyses in immuno-oncology, where heterogeneity in endpoint definitions was mitigated by focusing on their overlapping clinical significance (24, 25). Nevertheless, we acknowledge that subtle differences between EFS and DFS may influence the interpretation of results, particularly in neoadjuvant trials where EFS captures early therapeutic efficacy. In an ideal scenario, uniform endpoint reporting would enhance comparability; however, given the limited availability of data—especially in PD-L1 expression subgroups—pooling EFS and DFS provided a more comprehensive assessment of immunotherapy benefits across studies.

The recently reported CCTG BR.31 trial (26) showed that adjuvant durvalumab failed to improve DFS in completely resected IB-IIIA NSCLC, regardless of PD-L1 expression (≥25% or ≥1%). Despite these uncertainties, the Chinese expert consensus on perioperative immunotherapy for NSCLC recommends continuing adjuvant immunotherapy, particularly for patients who have received neoadjuvant treatment, based on the potential for additional survival benefits (21). Our findings support this recommendation, demonstrating EFS benefits across all PD-L1 subgroups, including PD-L1<1% patients. This suggests that adjuvant immunotherapy may improve outcomes even in patients with low or absent PD-L1 expression, especially those who do not achieve pCR or MPR after neoadjuvant therapy. Recent individual patient-level analyses from the CheckMate-816 and CheckMate-77T trials provide further insights. Compared to neoadjuvant nivolumab alone, perioperative nivolumab significantly improved EFS (HR=0.61, 95% CI: 0.39–0.97). Subgroup analyses revealed that patients who did not achieve pCR after neoadjuvant therapy derived greater EFS benefits from adjuvant treatment (HR=0.65, 95% CI: 0.40–1.06), whereas those who achieved pCR did not (HR=0.58, 95% CI: 0.14–2.40). Intriguingly, only PD-L1<1% patients showed significant EFS benefits from perioperative immunotherapy (HR=0.51, 95% CI: 0.28–0.93), while PD-L1≥1% patients did not (HR=0.86, 95% CI: 0.44–1.70). These findings suggest that while adjuvant immunotherapy may enhance outcomes, its benefits might be less pronounced in PD-L1-high patients, particularly those who achieve pCR after neoadjuvant therapy. This may indicate that neoadjuvant immunotherapy alone is sufficient for these patients, whereas those who do not achieve pCR may require additional postoperative interventions, including immunotherapy, regardless of PD-L1 status. However, the limited number of studies reporting OS outcomes in our meta-analysis underscores the need for long-term follow-up to confirm these findings.

The cumulative meta-analysis results show that the efficacy of immunotherapy improves over time. This likely reflect several key developments. Later trials might better identify likely responders through refined inclusion criteria and biomarker understanding, even for PD-L1<1% patients. Advances in surgical techniques, perioperative care, and irAE management also enable more patients to complete treatment successfully. Growing clinician experience and institutional protocols can improve immunotherapy delivery and toxicity management. While we cannot definitively determine causality, this consistent trend across outcomes suggests improvements in immunotherapy application. The findings support expanding perioperative immunotherapy use while continuing to optimize patient selection and treatment protocols.

Our findings emphasize the prognostic value of PD-L1 expression, yet the influence of histological subtypes (squamous versus non-squamous NSCLC) on immunotherapy efficacy remains incompletely defined. Although none of the included trials exclusively enrolled specific histological subtypes, we conducted exploratory subgroup analyses based on the proportion of squamous NSCLC within each study cohort. Notably, studies with ≥50% squamous NSCLC demonstrated consistently stronger EFS benefits across all PD-L1 expression strata compared to studies with <50% squamous NSCLC (**Table 2**). For example, in the PD-L1≥1% subgroup, the pooled HR for EFS was 0.485 (95% CI: 0.374–0.629) in studies enriched with squamous NSCLC versus 0.554 (95% CI: 0.458–0.670) in studies with fewer squamous cases. This trend aligns with emerging biological evidence suggesting distinct immune microenvironment features in squamous NSCLC, such as higher tumor mutational burden or enhanced antigen presentation, which may amplify immunotherapy responsiveness regardless of PD-L1 levels. However, only the IMpower010 trial reported PD-L1-stratified outcomes separately for squamous and non-squamous subtypes, precluding a robust pooled analysis. The scarcity of histological subtype-specific data represents a critical limitation. Future trials should integrate preplanned analyses stratifying outcomes by both PD-L1 expression and histological subtype to address this gap, ultimately advancing precision medicine in NSCLC.

The observed benefits in the PD-L1<1% subgroup might also be due to population heterogeneity, since a proportion of patients had significant benefits despite low PD-L1 expression. Future research should explore potential biomarkers beyond PD-L1, such as ctDNA and lymph node involvement (27), as well as some critical biomarkers revealed in advanced NSCLC such as TMB, driver gene mutations, and TME characteristics (28). These would help to better predict which patients are most likely to benefit from immunotherapy.

This meta-analysis has several limitations that warrant consideration. First, the assessment of OS, the critical endpoint for evaluating long-term survival benefits, was limited by the small number of studies reporting stratified OS outcomes across PD-L1 subgroups. Second, unlike individual clinical trials, meta-analyses cannot perform formal interaction tests to directly compare treatment effects across different PD-L1 expression levels. Instead, we relied on the magnitude of effect sizes (ORs and HRs) to infer the degree of benefit, which may introduce interpretative challenges. Third, the current evidence base remains limited, with more robust data available for perioperative approaches (n=6 studies) compared to neoadjuvant (n=2) and adjuvant (n=2) settings. This precluded definitive comparisons of PD-L1’s predictive utility between treatment modalities. These knowledge gaps highlight the need for future trials to incorporate uniform PD-L1 stratification thresholds across all treatment settings.

## 5 Conclusion

This meta-analysis demonstrates that immunotherapy significantly improves pCR, MPR, and EFS across all PD-L1 expression subgroups in resectable NSCLC, with the greatest benefits observed in PD-L1-high patients. While OS benefits are established in PD-L1≥1% populations, the impact on PD-L1<1% patients remains uncertain. The findings support the integration of immunotherapy into perioperative care, particularly for patients with higher PD-L1 expression, but also highlight its potential utility in PD-L1<1% subgroup. Future research should focus on clarifying the role of immunotherapy in neoadjuvant and adjuvant settings, optimizing patient selection, and addressing the limitations of currently available OS evidence.

## Data Availability

The original contributions presented in the study are included in the article/**Supplementary Material**. Further inquiries can be directed to the corresponding author.

## Supplementary material

Supplementary Figure 1 Sensitivity analysis of treatment outcomes in patients stratified by PD-L1 expression levels <1% and ≥1% This figure includes sensitivity analyses for key outcomes, comprising **(A)** pathological complete response (pCR) rate in patients with PD-L1 expression <1%, **(B)** major pathological response (MPR) rate in patients with PD-L1 expression <1%, **(C)** event-free survival (EFS) in patients with PD-L1 expression <1%, **(D)** overall survival (OS) in patients with PD-L1 expression <1%, **(E)** pCR rate in patients with PD-L1 expression ≥1%, **(F)** MPR rate in patients with PD-L1 expression ≥1%, **(G)** EFS in patients with PD-L1 expression ≥1%, and **(H)** OS in patients with PD-L1 expression ≥1%. Each forest plot shows the effect of omitting individual studies on the pooled estimates for the respective outcome measures.

Supplementary Figure 2 Sensitivity analysis of treatment outcomes in patients stratified by PD-L1 expression levels 1-49% and ≥50%. This figure includes sensitivity analyses for key outcomes, comprising **(A)** pathological complete response (pCR) rate in patients with PD-L1 expression between 1-49%, **(B)** major pathological response (MPR) rate in patients with PD-L1 expression between 1-49%, **(C)** event-free survival (EFS) in patients with PD-L1 expression between 1-49%, **(D)** overall survival (OS) in patients with PD-L1 expression between 1-49%, **(E)** pCR rate in patients with PD-L1 expression ≥50%, **(F)** MPR rate in patients with PD-L1 expression ≥50%, **(G)** EFS in patients with PD-L1 expression ≥50%, and (H) OS in patients with PD-L1 expression ≥50%. Each forest plot shows the effect of omitting individual studies on the pooled estimates for the respective outcome measures.

Supplementary Figure 3 Publication bias for event-free survival (EFS). Publication bias for EFS was assessed by funnel plot in patients with **(A)** PD-L1 expression <1%, **(B)** PD-L1 expression ≥1%, **(C)** PD-L1 expression between 1-49%, and **(D)** PD-L1 expression ≥50%.

Supplementary Table 1 Outcomes in neoadjuvant, perioperative, adjuvant setting. PD-L1, programmed death ligand 1; pCR, pathological complete response; MPR, major pathological response; EFS, event-free survival; OS, overall survival; OR, odds ratio; CI, confidence interval; NA, not applicable.
