# Supplementary figures and images for: Efficacy of neoadjuvant, adjuvant, and perioperative immunotherapy in non-small cell lung cancer across different PD-L1 expression levels: a systematic review and meta-analysis
Source: Front Immunol. 2025 May 20;16:1569864. doi: 10.3389/fimmu.2025.1569864 (PMC12129973; doi:10.3389/fimmu.2025.1569864)

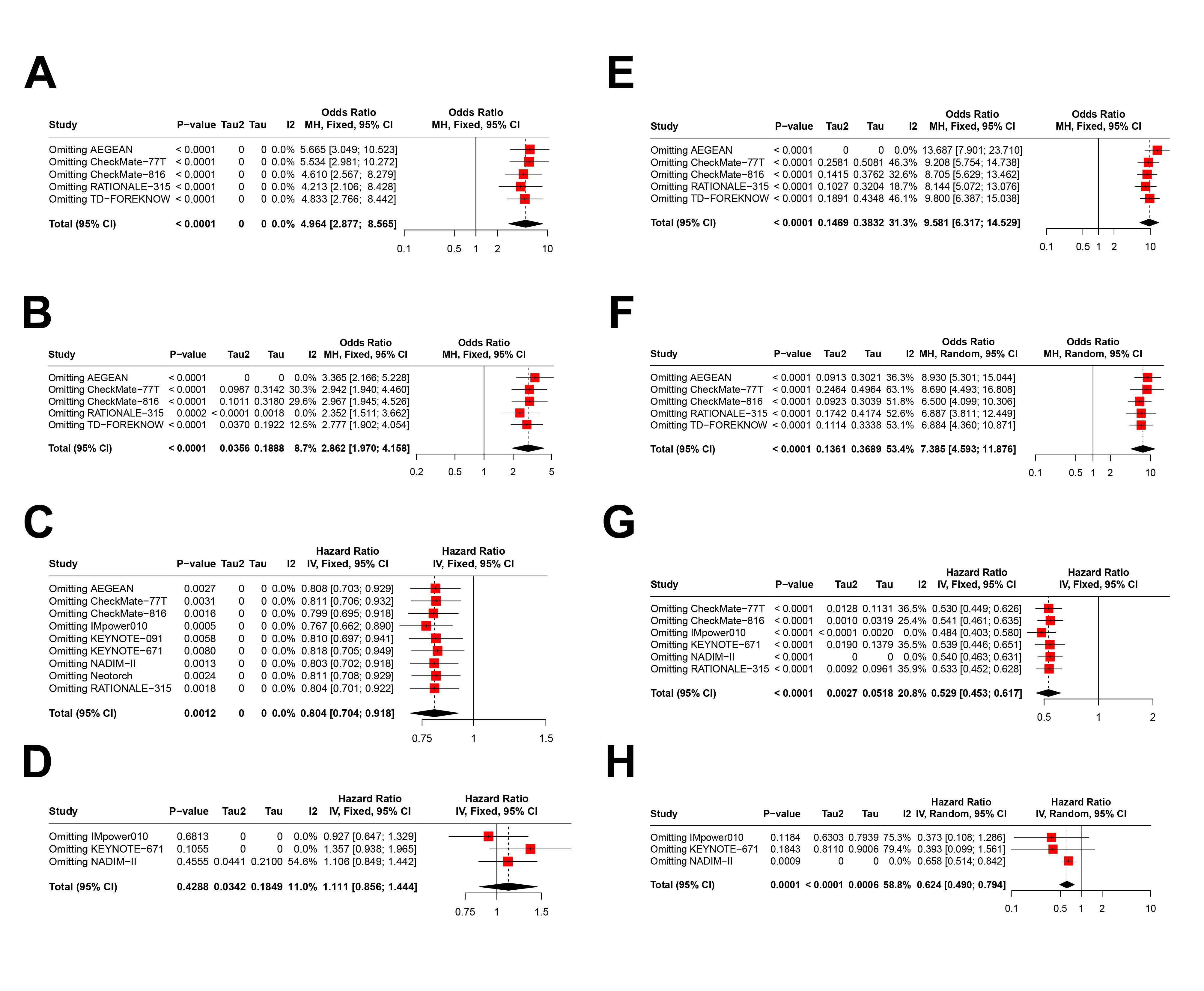

Supplement: Supplementary Figure 1 — Sensitivity analysis of treatment outcomes in patients stratified by PD-L1 expression levels <1% and ≥1% This figure includes sensitivity analyses for key outcomes, comprising (A) pathological complete response (pCR) rate in patients with PD-L1 expression <1%, (B) major pathological response (MPR) rate in patients with PD-L1 expression <1%, (C) event-free survival (EFS) in patients with PD-L1 expression <1%, (D) overall survival (OS) in patients with PD-L1 expression <1%, (E) pCR rate in patients with PD-L1 expression ≥1%, (F) MPR rate in patients with PD-L1 expression ≥1%, (G) EFS in patients with PD-L1 expression ≥1%, and (H) OS in patients with PD-L1 expression ≥1%. Each forest plot shows the effect of omitting individual studies on the pooled estimates for the respective outcome measures. [file Image1.tif]

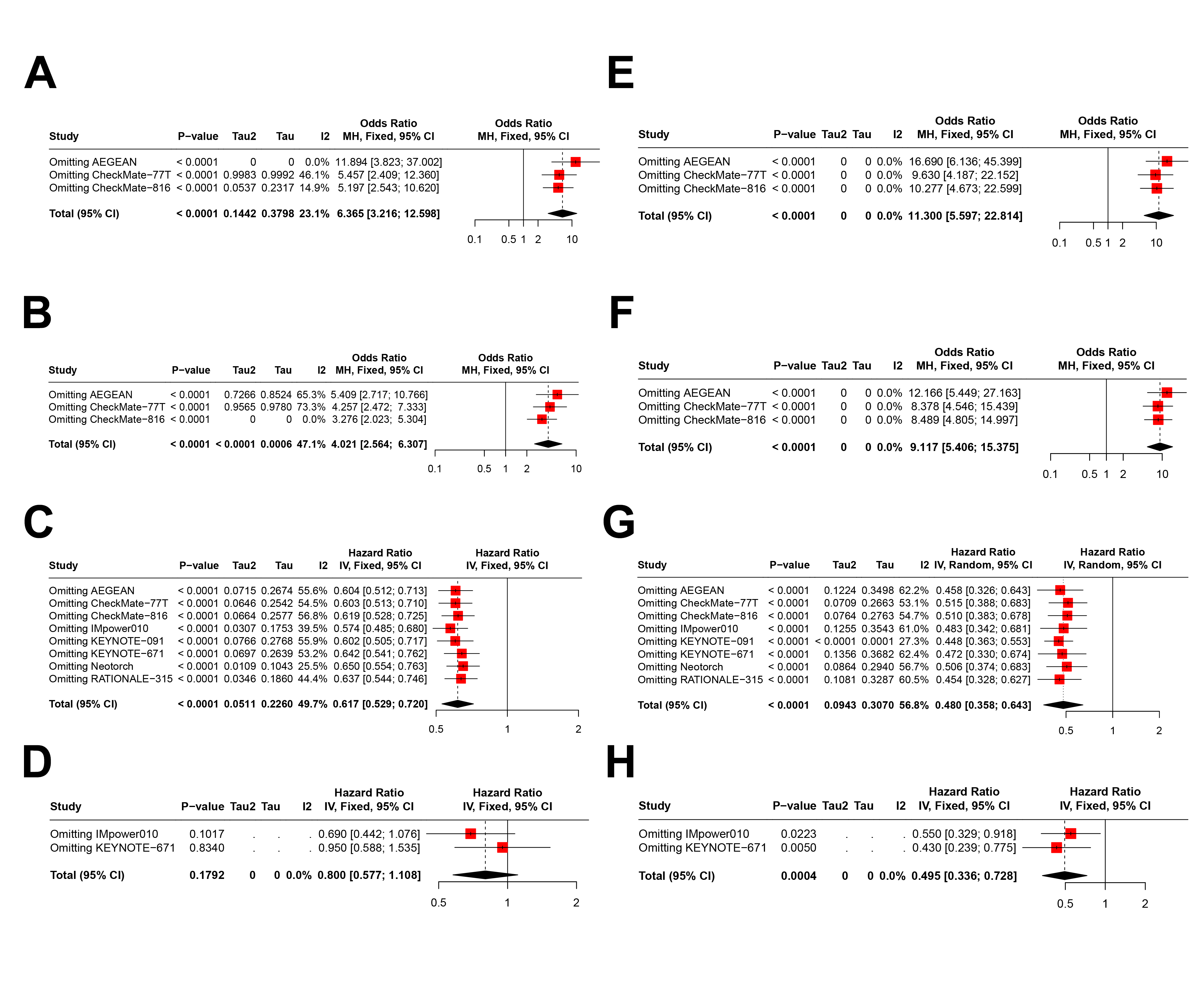

Supplement: Supplementary Figure 2 — Sensitivity analysis of treatment outcomes in patients stratified by PD-L1 expression levels 1-49% and ≥50%. This figure includes sensitivity analyses for key outcomes, comprising (A) pathological complete response (pCR) rate in patients with PD-L1 expression between 1-49%, (B) major pathological response (MPR) rate in patients with PD-L1 expression between 1-49%, (C) event-free survival (EFS) in patients with PD-L1 expression between 1-49%, (D) overall survival (OS) in patients with PD-L1 expression between 1-49%, (E) pCR rate in patients with PD-L1 expression ≥50%, (F) MPR rate in patients with PD-L1 expression ≥50%, (G) EFS in patients with PD-L1 expression ≥50%, and (H) OS in patients with PD-L1 expression ≥50%. Each forest plot shows the effect of omitting individual studies on the pooled estimates for the respective outcome measures. [file Image2.tif]

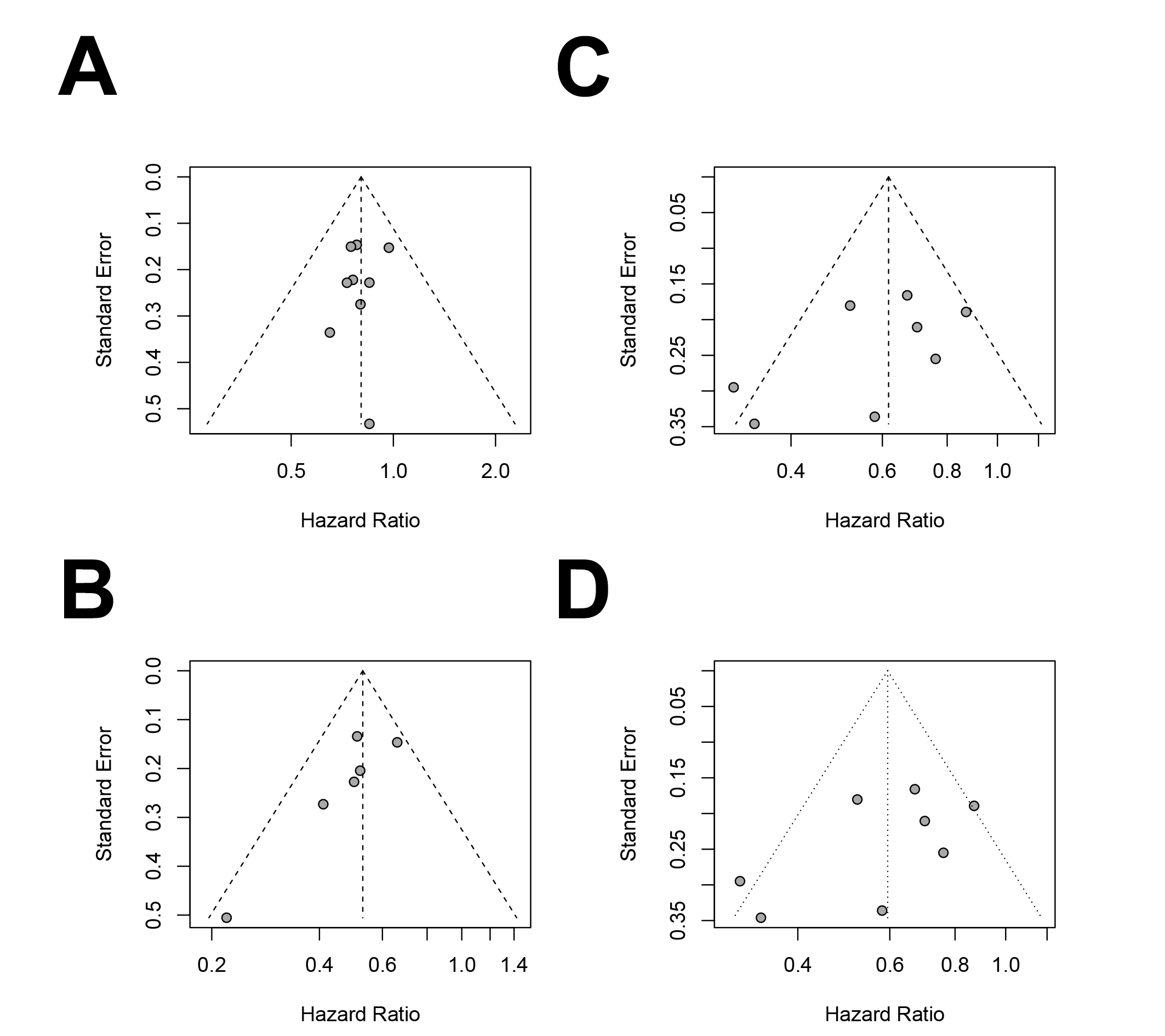

Supplement: Supplementary Figure 3 — Publication bias for event-free survival (EFS). Publication bias for EFS was assessed by funnel plot in patients with (A) PD-L1 expression <1%, (B) PD-L1 expression ≥1%, (C) PD-L1 expression between 1-49%, and (D) PD-L1 expression ≥50%. [file Image3.tif]
