# Supplementary material for: Efficacy of neoadjuvant, adjuvant, and perioperative immunotherapy in non-small cell lung cancer across different PD-L1 expression levels: a systematic review and meta-analysis
Source: Front Immunol. 2025 May 20;16:1569864. doi: 10.3389/fimmu.2025.1569864 (PMC12129973; doi:10.3389/fimmu.2025.1569864)
Supplement: Supplementary Table 1 — Outcomes in neoadjuvant, perioperative, adjuvant setting. PD-L1, programmed death ligand 1; pCR, pathological complete response; MPR, major pathological response; EFS, event-free survival; OS, overall survival; OR, odds ratio; CI, confidence interval; NA, not applicable. [file Table1.docx]

**Supplementary Table S1. Outcomes in neoadjuvant, perioperative, adjuvant setting**

| Immunotherapy setting and PD-L1 stratification | pCR | | MPR | | EFS | | OS | |
| --- | --- | --- | --- | --- | --- | --- | --- | --- |
|  | No. of studies | OR and 95%CI | No. of studies | OR and 95%CI | No. of studies | HR and 95%CI | No. of studies | HR and 95%CI |
| Neoadjuvant |  |  |  |  |  |  |  |  |
| PD-L1<1% | 2 | 7.853 (2.089-29.520) | 2 | 2.874 (1.346-6.136) | 1 | 0.850 (0.544-1.329) | 0 | NA |
| PD-L1≥1% | 2 | 14.400 (4.699-44.124) | 2 | 15.678 (6.241-39.383) | 1 | 0.410 (0.240-0.700) | 0 | NA |
| PD-L1: 1-49% | 1 | 30.063 (1.725-523.920) | 1 | 15.750 (3.437-72.171) | 1 | 0.580 (0.300-1.121) | 0 | NA |
| PD-L1≥50% | 1 | 16.191 (3.411-76.852) | 1 | 13.000 (3.420-49.421) | 1 | 0.240 (0.097-0.593) | 0 | NA |
| Perioperative |  |  |  |  |  |  |  |  |
| PD-L1<1% | 3 | 4.447 (2.438-8.114) | 3 | 2.858 (1.860-4.390) | 6 | 0.749 (0.621-0.903) | 2 | 0.927 (0.647-1.329) |
| PD-L1≥1% | 3 | 8.888 (5.671-13.930) | 3 | 5.871 (4.304-8.008) | 4 | 0.494 (0.407-0.600) | 2 | 0.373 (0.108-1.286) |
| PD-L1: 1-49% | 2 | 5.197 (2.543-10.620) | 2 | 3.276 (2.024-5.304) | 5 | 0.518 (0.374-0.717) | 1 | 0.690 (0.443-1.076) |
| PD-L1≥50% | 2 | 10.277 (4.673-22.599) | 2 | 8.489 (4.805-14.997) | 5 | 0.475 (0.372-0.606) | 1 | 0.550 (0.329-0.918) |
| Adjuvant |  |  |  |  |  |  |  |  |
| PD-L1<1% | NA | NA | NA | NA | 2 | 0.866 (0.704-1.065) | 1 | 1.360 (0.930-1.989) |
| PD-L1≥1% | NA | NA | NA | NA | 1 | 0.660 (0.495-0.879) | 1 | 0.710 (0.490-1.029) |
| PD-L1: 1-49% | NA | NA | NA | NA | 2 | 0.751 (0.588-0.959) | 1 | 0.950 (0.588-1.535) |
| PD-L1≥50% | NA | NA | NA | NA | 2 | 0.604 (0.321-1.135) | 1 | 0.430 (0.239-0.775) |

Abbreviations: PD-L1, programmed death ligand 1; pCR, pathological complete response; MPR, major pathological response; EFS, event-free survival; OS, overall survival; OR, odds ratio; CI, confidence interval; NA, not applicable.
